# Sofpironium Bromide for the Treatment of Primary Axillary Hyperhidrosis: A Systematic Review and Meta-Analysis of Randomized Controlled Trials

**DOI:** 10.7759/cureus.108570

**Published:** 2026-05-09

**Authors:** Devanie Martani, Milena S Silva, Luciana Nakanishi, Laís F Valente, Georgina G Garza-Dueñas, Meire O Silva, Elisete I Crocco

**Affiliations:** 1 Faculty of Medicine, Universitas Tarumanagara, Jakarta, IDN; 2 Faculty of Medicine, Faculdade de Ciências Médicas de Santos, Santos, BRA; 3 Dermatology, Clínica Concept, Mogi das Cruzes, BRA; 4 Faculty of Medicine, Faculdade Pernambucana de Saúde, Recife, BRA; 5 Faculty of Medicine, Universidad Autonoma de Nuevo León (UANL), Monterrey, MEX; 6 Faculty of Medicine, Universidad del Pacifico, Pedro Juan Caballero, PRY; 7 Faculty of Medical Sciences, Santa Casa de São Paulo, São Paulo, BRA

**Keywords:** cholinergic antagonists, hyperhidrosis, meta-analysis, sofpironium bromide, topical administration

## Abstract

Sofpironium bromide is a recently approved topical anticholinergic agent for primary axillary hyperhidrosis, developed using a retrometabolic approach to limit systemic side effects. However, randomized evidence about its effects has not yet been quantitatively synthesized. This meta-analysis aimed to assess the efficacy and safety of topical sofpironium bromide compared with a vehicle in patients with primary axillary hyperhidrosis. PubMed, Embase, Cochrane Central Register of Controlled Trials (CENTRAL), and ClinicalTrials.gov were searched for randomized controlled trials (RCTs) comparing topical sofpironium bromide with a vehicle. Risk ratios (RRs) and mean differences (MDs) with 95% confidence intervals (CIs) were pooled using random-effects models in Review Manager 7.2.0 (The Cochrane Collaboration, London, UK). Five RCTs, including 1,398 participants, were included, of whom 807 (57.7%) received topical sofpironium bromide. Compared with vehicle, sofpironium bromide significantly reduced gravimetric sweat production (GSP) at the end of treatment (MD -25.27 mg; 95% CI -40.15 to -10.40) and increased the likelihood of achieving at least a two-point improvement in the Hyperhidrosis Disease Severity Scale (HDSS) (RR 3.27; 95% CI 1.57-6.80). Discontinuation due to adverse events was more frequent in the sofpironium bromide group (RR 9.83; 95% CI 1.82-53.17). Adverse events occurred more frequently in the treatment group, including application-site dermatitis (RR 5.26; 95% CI 2.05-13.48), application-site pain (RR 4.63; 95% CI 2.08-10.28), pruritus (RR 5.97; 95% CI 1.98-18.02), and dry mouth (RR 15.45; 95% CI 5.35-44.62). No significant increase in serious adverse events was observed. Overall, this meta-analysis supports the efficacy of sofpironium bromide for primary axillary hyperhidrosis, while treatment decisions should be weighed against potential side effects.

## Introduction and background

Hyperhidrosis is a condition characterized by excessive sweating beyond the physiological needs of thermoregulation, caused by overactivity of the eccrine sweat glands. It can be classified as primary or secondary, with its pathophysiology closely linked to the sympathetic nervous system [1]. Although the exact etiology remains unclear, genetic predisposition appears to play a role in primary hyperhidrosis, which accounts for the majority of cases [1,2]. Reported prevalence varies widely, ranging from approximately 0.9% in Brazil to 4.8% in the United States, with even higher rates observed among specific groups such as university students [3-6]. This condition can significantly impair patients’ quality of life, particularly in social and professional settings, leading to psychosocial distress and reduced well-being [7].

Current therapeutic approaches are usually selected according to symptom severity, anatomic site, and patient response [8]. Oral anticholinergics such as oxybutynin and glycopyrrolate are commonly used, although adverse effects such as dry mouth, blurred vision, and urinary retention may limit continued use [8]. Other procedures, including botulinum toxin injections, might not be suitable for all patients due to discomfort and need for repeated treatments, while sympathectomy is an invasive procedure that carries surgical risks and may result in compensatory hyperhidrosis [8,9].

Consequently, topical therapies have become preferred for managing primary hyperhidrosis. Among these, sofpironium bromide was approved by the U.S. Food and Drug Administration (FDA) in June 2024 for this indication [10]. This topical anticholinergic was developed to reduce excessive sweating while minimizing systemic adverse effects. To date, five randomized controlled trials (RCTs) have evaluated its efficacy and safety in primary axillary hyperhidrosis. However, to our knowledge, no meta-analysis has quantitatively synthesized the available evidence. Therefore, this study aims to provide a comprehensive assessment of current data to better understand the therapeutic role of sofpironium bromide in the management of primary axillary hyperhidrosis.

## Review

Methods 

This systematic review and meta-analysis was conducted in accordance with the Cochrane Handbook for Systematic Reviews of Interventions and reported following the Preferred Reporting Items for Systematic Reviews and Meta-Analyses (PRISMA) 2020 guidelines [11,12]. The protocol was registered on the International Prospective Register of Systematic Reviews (PROSPERO) under the registration number CRD420251125720.

Eligibility Criteria 

We included studies involving patients with primary axillary hyperhidrosis that used an RCT design, compared sofpironium bromide with vehicle, and reported at least one outcome of interest. No language restrictions were applied. We excluded observational studies, case reports, case series, reviews, commentaries, editorials, abstracts, letters, and animal studies due to their greater susceptibility to bias compared to RCTs. Their inclusion may also increase heterogeneity, potentially affecting the reliability of the pooled results. Studies were also excluded if they involved overlapping populations, defined as multiple publications reporting results from the same or partially overlapping patient cohorts, in order to avoid duplication of data, or if they focused on conditions other than primary axillary hyperhidrosis.

Search Strategy and Data Extraction

We systematically searched PubMed, Embase, the Cochrane Central Register of Controlled Trials (CENTRAL), and ClinicalTrials.gov for RCTs from inception to July 2025. The search strategy combined relevant keywords and synonyms related to hyperhidrosis and sofpironium bromide. The full search strategies for all databases are provided in Appendix 1. Articles retrieved from the database search were imported into Zotero (Corporation for Digital Scholarship, Vienna, VA), where duplicates were removed, and the remaining records were screened. Two authors independently screened eligible RCTs and extracted data. Any disagreements were resolved by consensus with a senior author of this paper.

Endpoints and Subgroup Analysis

The primary outcome was gravimetric sweat production (GSP), as it represents an objective measure of sweat reduction. Secondary outcomes included the Hyperhidrosis Disease Severity Scale (HDSS), Hyperhidrosis Disease Severity Measure-Axillary (HDSM-Ax), and Dermatology Life Quality Index (DLQI), which reflect symptom severity and quality of life. Safety outcomes included the incidence of any reported adverse events. All outcomes were assessed at the end of the treatment period. Subgroup analyses were performed for the GSP outcome based on treatment duration and sofpironium bromide dosage.

Statistical Analysis

Risk ratios (RRs) were calculated for binary outcomes, and mean differences (MDs) for continuous outcomes, both with 95% confidence intervals (CIs). The generic inverse variance method was applied when studies reported adjusted estimates, such as least squares means. Heterogeneity was assessed using the Cochran Q test and the I^2^ statistic, with P < 0.10 and I² > 25% indicating significant heterogeneity. A random-effects model using restricted maximum likelihood (REML) estimation was applied. The Hartung-Knapp-Sidik-Jonkman (HKSJ) method was used to calculate CIs when ≥3 studies were available, and heterogeneity was greater than zero; otherwise, the Wald-type method was applied [12]. A leave-one-out sensitivity analysis was performed for outcomes with substantial heterogeneity (I² > 50%) to evaluate the robustness of pooled estimates. All statistical analyses were performed using Review Manager Version 7.2.0 (The Cochrane Collaboration, London, UK).

Quality Assessment

Each study included was assessed for risk of bias using the Cochrane Collaboration’s tool for assessing risk of bias in randomized trials (RoB 2). The RoB 2 tool evaluates five domains: bias arising from the randomization process; bias due to deviations from intended interventions; bias due to missing outcome data; bias in measurement of the outcome; and bias in selection of the reported result. Based on these domains, studies were categorized as high risk, some concerns, or low risk of bias. Two authors independently evaluated the risk of bias, and any disagreements were resolved in consultation with a senior author.

Results

Study Selection and Baseline Characteristics 

**Figure 1:**
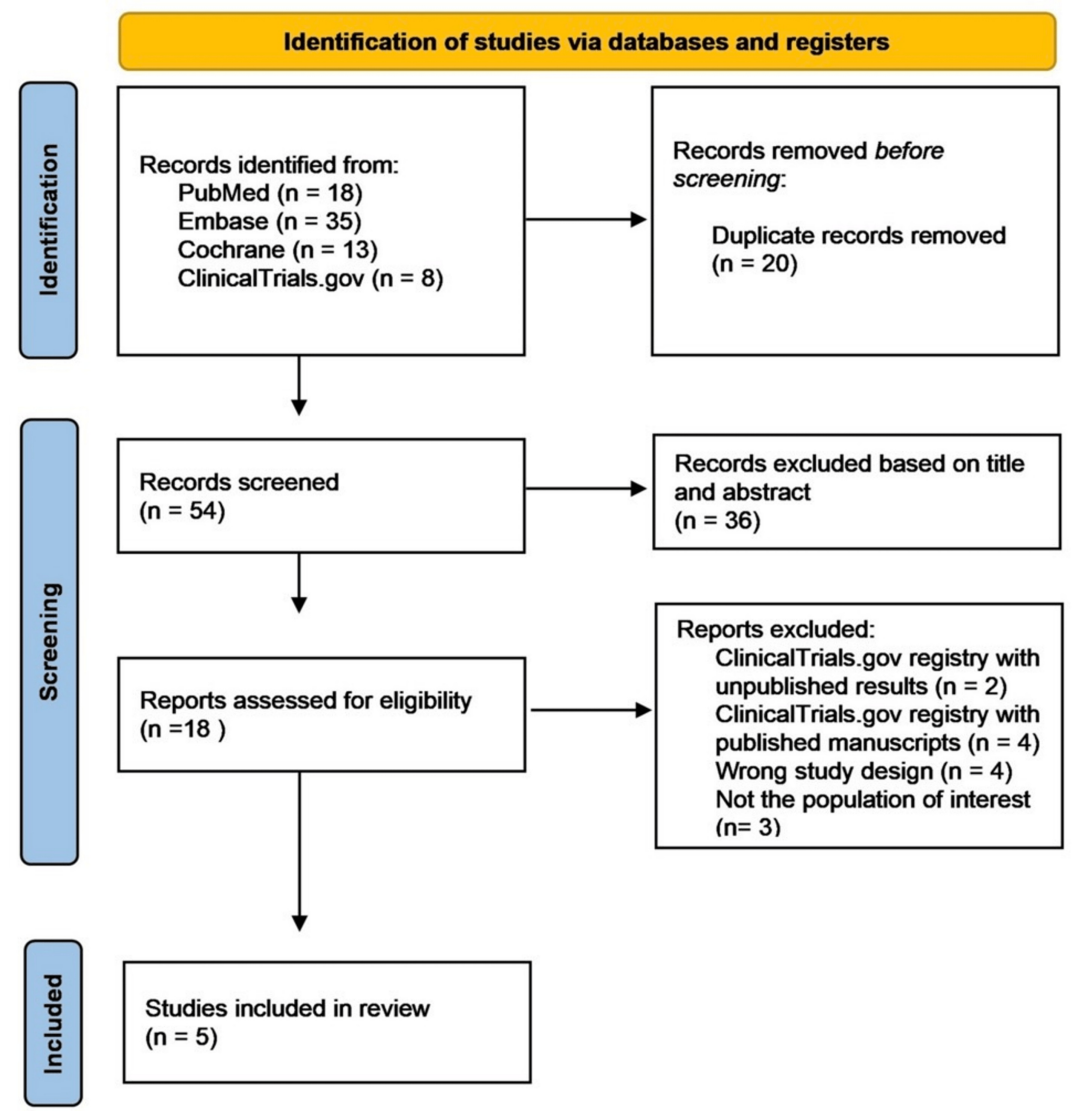
PRISMA flow diagram of study screening and selection PRISMA: Preferred Reporting Items for Systematic Reviews and Meta-Analyses

The initial search yielded 74 results. After removing duplicate records and ineligible studies, 18 remained and were fully reviewed based on eligibility criteria. A total of five RCTs comprising 1,398 participants were included in the analysis [13-16]. Among them, 807 participants (57.7%) received sofpironium bromide therapy. The study selection process is detailed in Figure 1. The included studies were phase 2 and phase 3 RCTs, with treatment durations of six weeks in four studies and four weeks in one study. The baseline characteristics of the included studies are presented in Table 1. 

**Table 1:** Baseline characteristics of included studies HDSM-Ax: Hyperhidrosis Disease Severity Measure-Axillary; GSP: gravimetric sweat production; SD: standard deviation ^a^HDSM-Ax-7

Study	Phase	Intervention	Patients (n)	Female, n (%)	Age (years), mean (SD)	Ethnicity (Hispanic), n (%)	Race (White), n (%)	HDSM-Ax, mean (SD)	GSP (mg), mean (SD)	Treatment duration
Kirsch et al. (2020) [13]	Phase 2	Sofpironium bromide 5 %	57	25 (44)	30.8 (10.2)	8 (14)	59 (86)	3.49 (0.32)	274.3 (191.4)	6 weeks
		Sofpironium bromide 10 %	57	22 (39)	33.7 (11.3)	12 (21)	45 (79)	3.50 (0.29)	288.5 (195.6)	
		Sofpironium bromide 15 %	56	25 (46)	30.7 (9.2)	15 (28)	42 (78)	3.57 (0.31)	311.1 (187.2)	
		Vehicle	57	30 (53)	30.0 (8.6)	13 (23)	43 (75)	3.39 (0.29)	279.4 (178.8)	
NCT02336503 [14]	Phase 2	BBI-4000 Gel, 5 %	47	24 (51.1)	32.6 (10.34)	13 (27.7)	35 (74.5)	NA	NA	4 weeks
		BBI-4000 Gel, 10%	48	25 (52.1)	33.4 (10.61)	12 (25)	34 (70.8)	NA	NA	
		BBI-4000 Gel, 15%	48	27 (56.3)	35.5 (11.64)	11 (22.9)	35 (72.9)	NA	NA	
		Vehicle	46	27 (58.7)	32.4 (8.23)	13 (28.3)	32 (69.6)	NA	NA	
Yokozeki et al. (2021) [15]	Phase 3	Sofpironium bromide 5 %	141	98 (69.5)	35.6 (13.44)	NA	NA	3.06 (0.514)	228 (167.10)	6 weeks
		Vehicle	140	99 (70.7)	36.1 (12.44)	NA	NA	3.04 (0.572)	226.3 (128.88)	
CARDIGAN I (2025) [16,17]	Phase 3	Sofpironium bromide 15 %	173	98 (56.6)	32.9 (11.63)	61 (35.3)	140 (80.9)	3.5 (0.3)^a^	296.2 (257.1)	6 weeks
		Vehicle	177	99 (55.9)	32.4 (10.86)	60 (33.9)	130 (73.4)	3.5 (0.3)^a^	298.5 (223.4)	
CARDIGAN II (2025) [16,17]	Phase 3	Sofpironium bromide 15 %	180	92 (51.1)	32.1 (12.22)	48 (26.7)	141 (78.3)	3.6 (0.3)^a^	313.8 (283.7)	6 weeks
		Vehicle	171	103 (60.2)	31.7 (11.13)	45 (26.3)	133 (77.8)	3.6 (0.3)^a^	308.4 (267.2)	

Outcome data were primarily extracted from published articles. For one study (NCT02336503), data were obtained from ClinicalTrials.gov. To minimize the risk of bias arising from missing evidence, supplementary information was retrieved from reliable alternative sources, including FDA multi-disciplinary reviews and trial registries, in accordance with Cochrane recommendations [12]. Data from multiple sources (e.g., FDA reports and published articles) were cross-checked using trial characteristics, including study name, study design, sample size, intervention, duration, and outcomes. In the forest plots, ‘CARDIGAN’ refers to the pooled result of the CARDIGAN I and CARDIGAN II trials.

Pooled Analysis of All Studies

**Figure 2:**
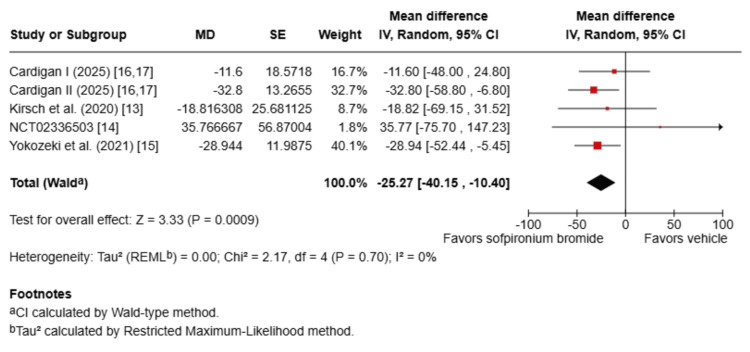
Forest plot comparing sofpironium bromide with vehicle for GSP at the end of treatment CI: confidence interval; IV: inverse variance; MD: mean difference; SE: standard error; GSP: gravimetric sweat production [13-17]

**Figure 3:**
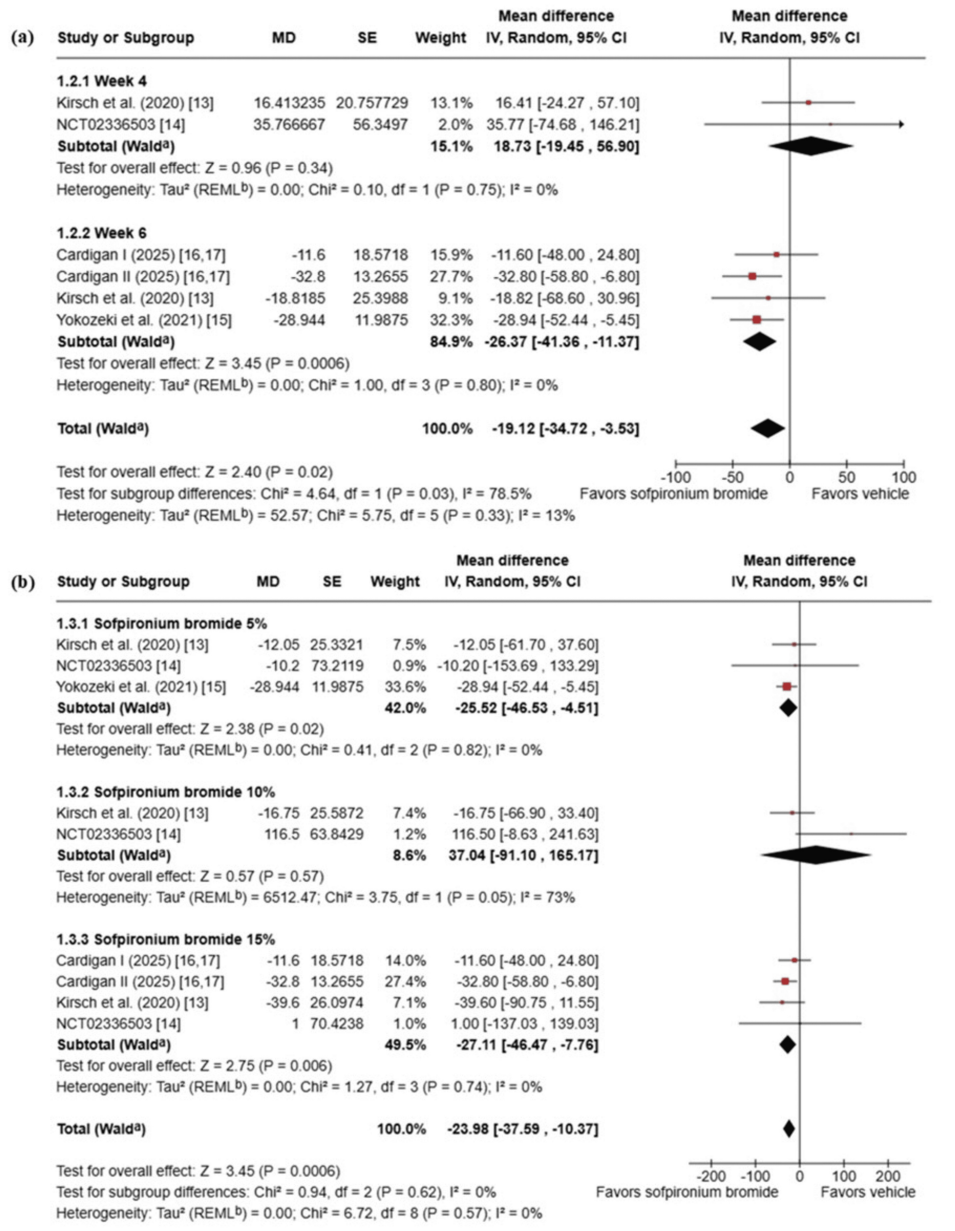
Forest plot comparing sofpironium bromide with vehicle for GSP subgroup analyses (a) by treatment duration (week four vs. week six) and (b) by treatment dosage MD: mean difference; SE: standard error; CI: confidence interval; IV: inverse variance; GSP: gravimetric sweat production [13-17]

A significant reduction in GSP was observed in the sofpironium bromide group compared with vehicle at the end of treatment (MD -25.27 mg; 95% CI -40.15 to -10.40; p = 0.0009; I² = 0%; Figure 2). To examine the consistency of this effect across different time points, we performed a subgroup analysis comparing week four and week six outcomes (Figure 3a). The results showed a significant improvement in the sofpironium bromide group at week six (MD -26.37 mg; 95% CI -41.36 to -11.37; p = 0.0006; I² = 0%), whereas no significant difference was observed at week four (MD 18.73 mg; 95% CI -19.45 to 56.90; p = 0.34; I² = 0%).

Subgroup analysis by dosage showed that both 5% and 15% sofpironium bromide groups significantly reduced GSP compared with vehicle, whereas the 10% group did not show a significant difference (Figure 3b). Pooled MD was -25.52 mg for the 5% group (95% CI -46.53 to -4.51; p = 0.02; I² = 0%), 37.04 mg for the 10% group (95% CI -91.10 to 165.17; p = 0.57; I² = 73%), and -27.11 mg for the 15% group (95% CI -46.47 to -7.76; p = 0.006; I² = 0%). There was no significant subgroup difference among the three dosage groups (p = 0.62; I² = 0%).

**Figure 4:**
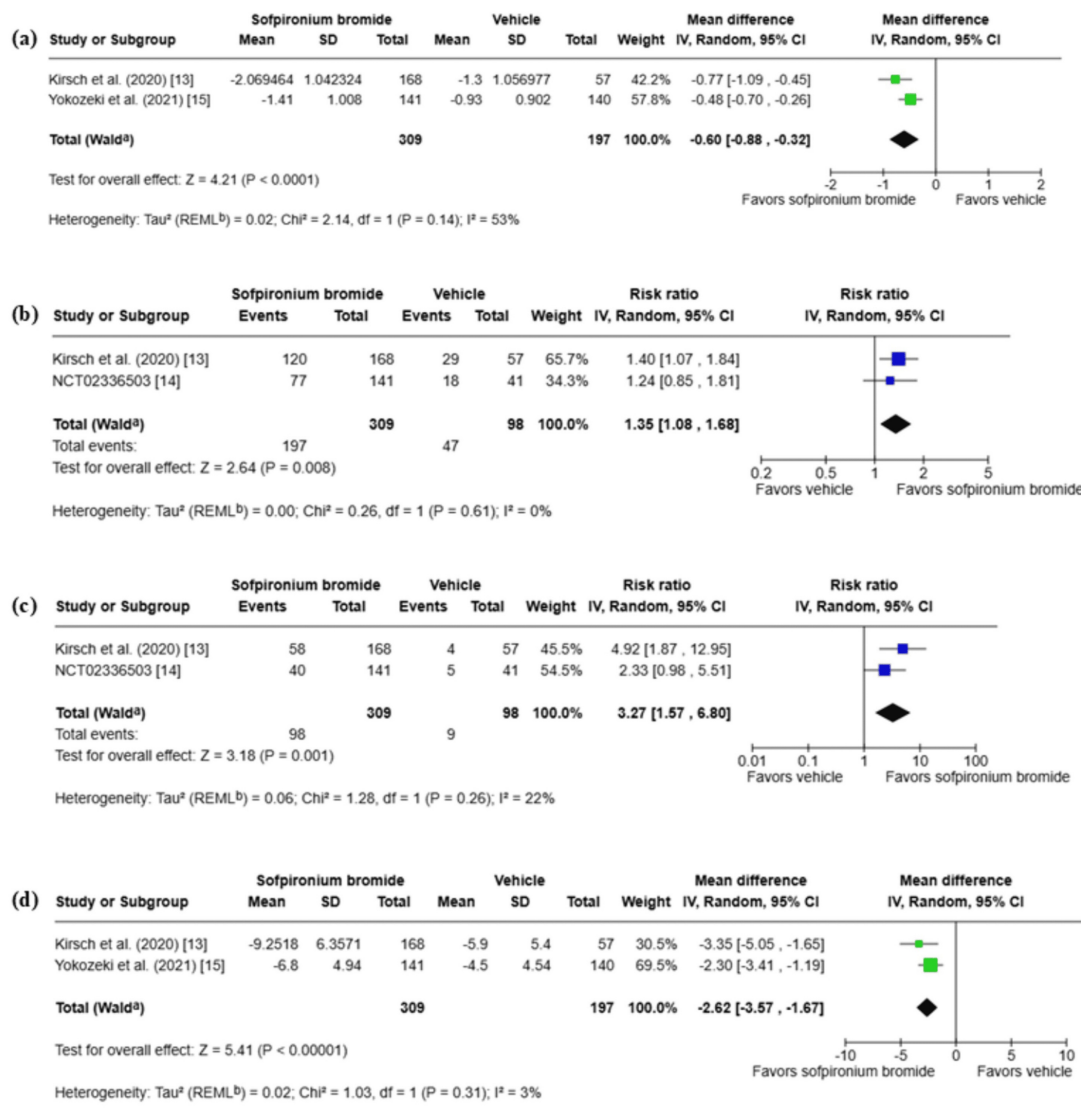
Forest plot comparing sofpironium bromide with vehicle for secondary outcomes (a) HDSM-Ax score, (b) at least a one-point improvement in HDSS, (c) at least a two-point improvement in HDSS, and (d) DLQI score CI: confidence interval; IV: inverse variance; SD: standard deviation; HDSM-Ax: Hyperhidrosis Disease Severity Measure-Axillary; HDSS: Hyperhidrosis Disease Severity Scale; DLQI: Dermatology Life Quality Index [13-17]

There was a greater reduction in HDSM-Ax scores in the sofpironium bromide group compared with vehicle (MD -0.60; 95% CI -0.88 to -0.32; p < 0.0001; I² = 53%; Figure 4a). Patients receiving topical sofpironium bromide were more likely to achieve at least a one-point improvement in HDSS compared with those receiving vehicle (RR 1.35; 95% CI 1.08-1.68; p = 0.008; I² = 0%; Figure 4b). Similarly, achieving at least a two-point improvement in HDSS was significantly more likely with sofpironium bromide (RR 3.27; 95% CI 1.57-6.80; p = 0.001; I² = 22%; Figure 4c). In addition, a significant improvement in DLQI score was observed in the sofpironium bromide group (MD -2.62; 95% CI -3.57 to -1.67; p < 0.00001; I² = 3%; Figure 4d).

**Figure 5:**
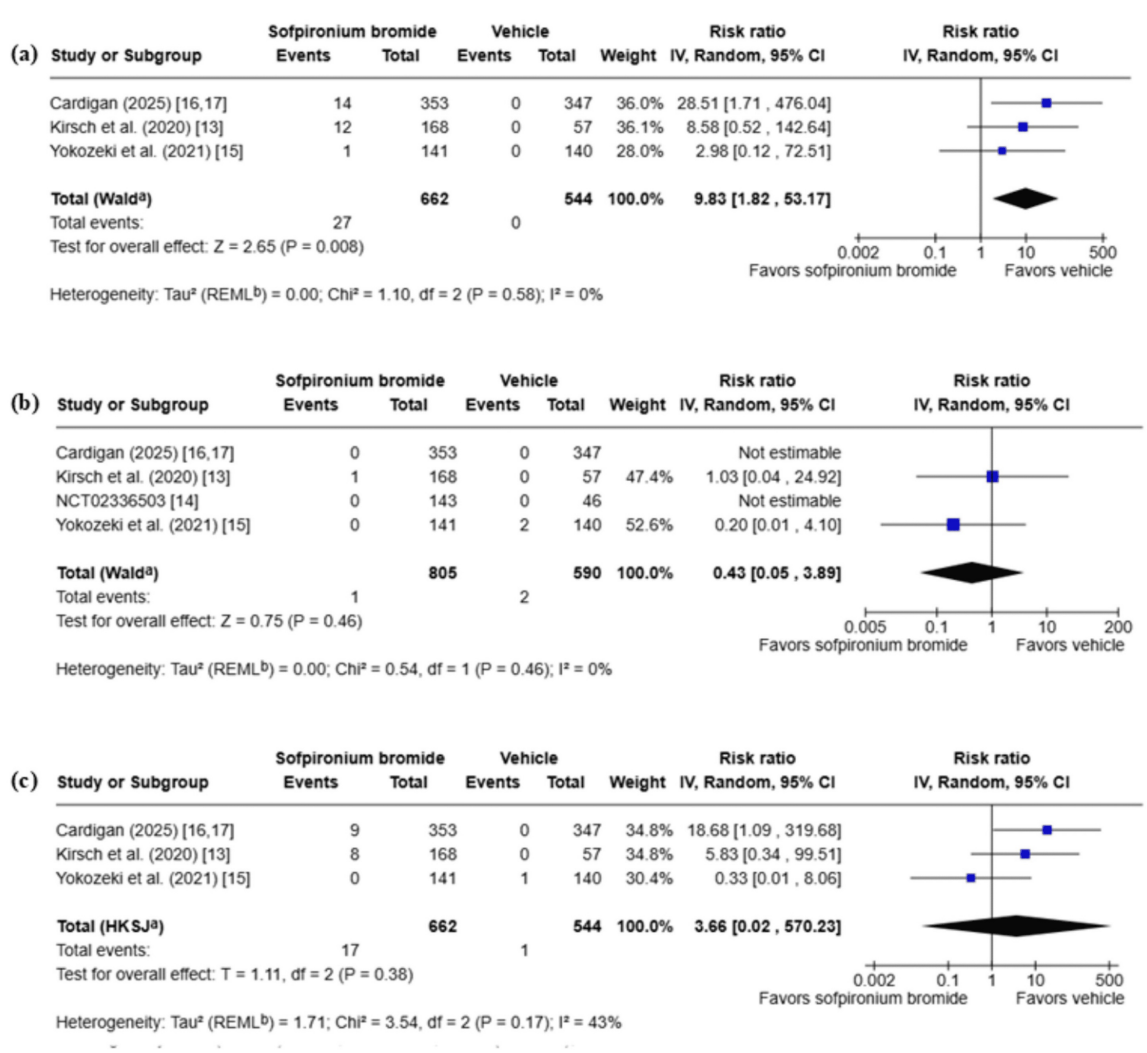
Forest plot comparing sofpironium bromide with vehicle for adverse events (a) discontinuation due to adverse events, (b) serious adverse events, and (c) severe adverse events CI: confidence interval; IV: inverse variance [13-17]

Discontinuation due to adverse events occurred more frequently in the sofpironium bromide group than in the vehicle group (RR 9.83; 95% CI 1.82-53.17; p = 0.008; I² = 0%; Figure 5a). There was no significant difference in the risk of serious adverse events between the two groups (RR 0.43; 95% CI 0.05-3.89; p = 0.46; I² = 0%; Figure 5b). Similarly, there was no significant difference in the risk of severe adverse events between the two groups (RR 3.66; 95% CI 0.02-570.23; p = 0.38; I² = 43%; Figure 5c). 

**Figure 6:**
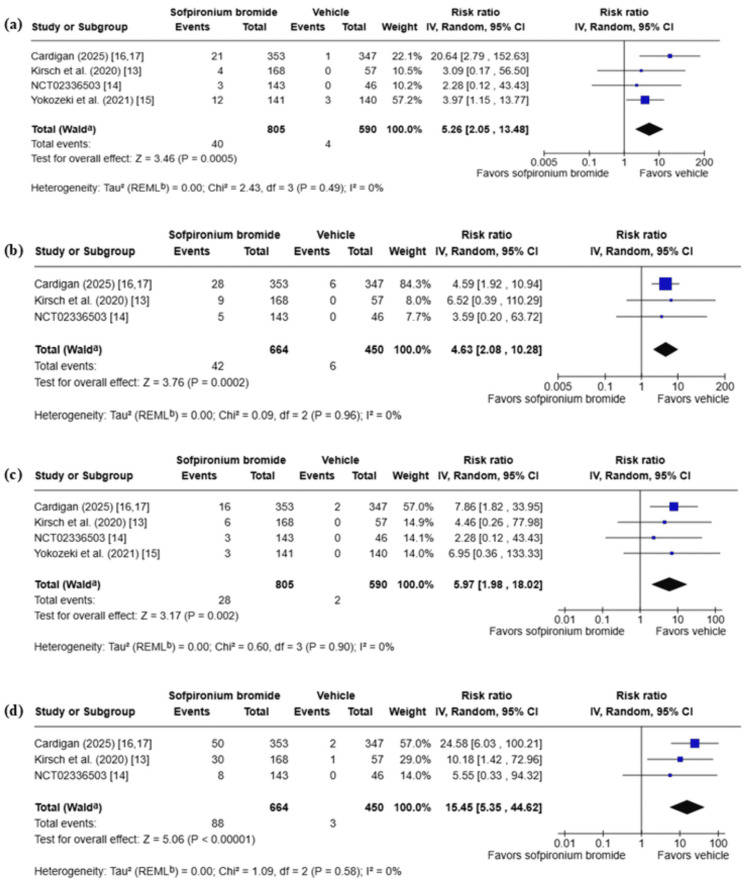
Forest plot comparing sofpironium bromide with vehicle for adverse events (a) application-site dermatitis, (b) application-site pain, (c) application-site pruritus, and (d) dry mouth CI: confidence interval; IV: inverse variance [13-17]

**Figure 7:**
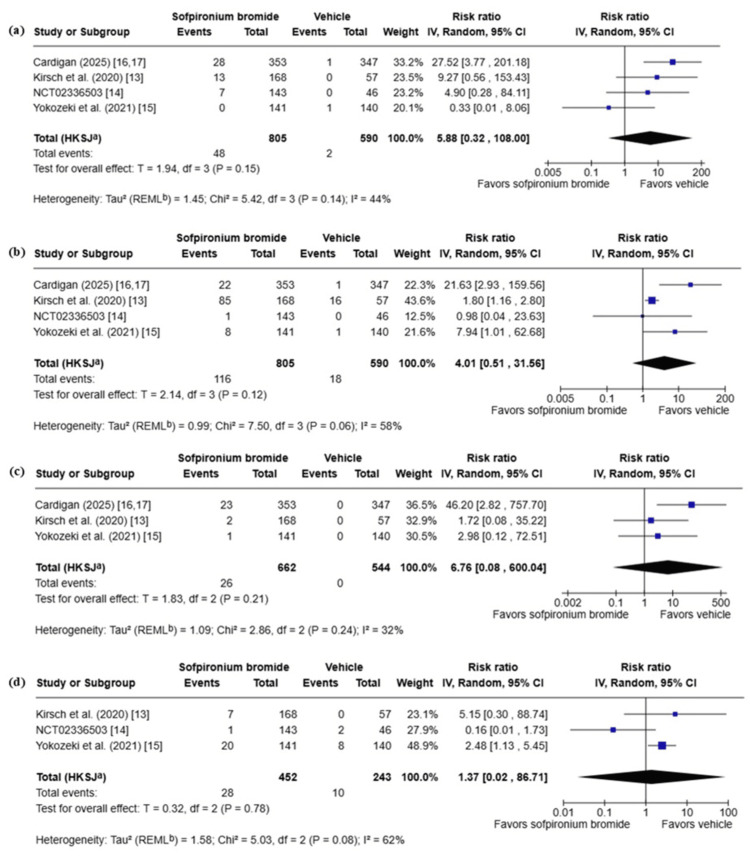
Forest plot comparing sofpironium bromide and vehicle for adverse events (a) blurred vision, (b) erythema, (c) mydriasis, and (d) nasopharyngitis CI: confidence interval; IV: inverse variance [13-17]

Adverse events were more frequent in the sofpironium bromide group. The most commonly reported events were application-site dermatitis (RR 5.26; 95% CI 2.05-13.48; p = 0.0005; I² = 0%; Figure 6a), application-site pain (RR 4.63; 95% CI 2.08-10.28; p = 0.0002; I² = 0%; Figure 6b), application-site pruritus (RR 5.97; 95% CI 1.98-18.02; p = 0.002; I² = 0%; Figure 6c), and dry mouth (RR 15.45; 95% CI 5.35-44.62; p < 0.00001; I² = 0%; Figure 6d). In contrast, no significant differences were found between groups in the incidence of blurred vision (RR 5.88; 95% CI 0.32-108.00; p = 0.15; I² = 44%; Figure 7a), erythema (RR 4.01; 95% CI 0.51-31.56; p = 0.12; I² = 58%; Figure 7b), mydriasis (RR 6.76; 95% CI 0.08-600.04; p = 0.21; I² = 32%; Figure 7c), or nasopharyngitis (RR 1.37; 95% CI 0.02-86.71; p = 0.78; I² = 62%; Figure 7d).

**Figure 8:**
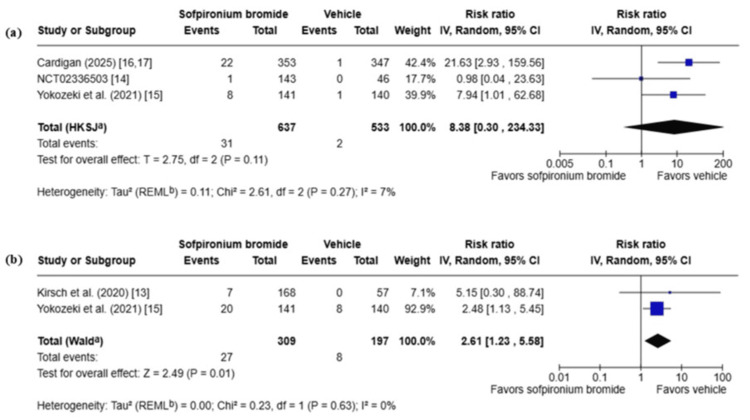
Leave-one-out sensitivity analysis forest plots (a) erythema and (b) nasopharyngitis CI: confidence interval; IV: inverse variance [13-17]

Due to high heterogeneity in erythema and nasopharyngitis outcomes, a leave-one-out sensitivity analysis was performed. Omitting the Kirsch et al. study from the erythema analysis reduced heterogeneity from I² = 58% to I² = 7% (RR 8.38; 95% CI 0.30-234.33; p = 0.11; Figure 8a). After omitting NCT02336503 from the nasopharyngitis analysis, heterogeneity decreased from I² = 62% to I² = 0% (RR 2.61; 95% CI 1.23-5.58; p = 0.01; Figure 8b). 

Risk of Bias Assessment

**Figure 9:**
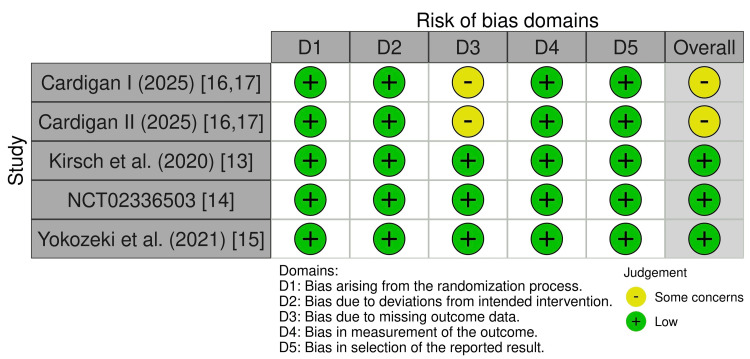
Risk of bias assessment [13-17]

Quality assessment was conducted using the Cochrane RoB 2 tool. Most included studies were rated as low risk of bias across domains. However, CARDIGAN I and CARDIGAN II were judged to have some concerns due to missing outcome data, resulting in an overall rating of some concerns for these two trials (Figure 9).

Discussion 

In this meta-analysis of five RCTs, we compared sofpironium bromide with vehicle. Our main findings were that the sofpironium bromide group had a significant reduction in GSP; a significant improvement in HDSS, HDSM-Ax, and DLQI scores; and more frequent adverse events, including application-site dermatitis, pain, pruritus, and dry mouth.

**Figure 10:**
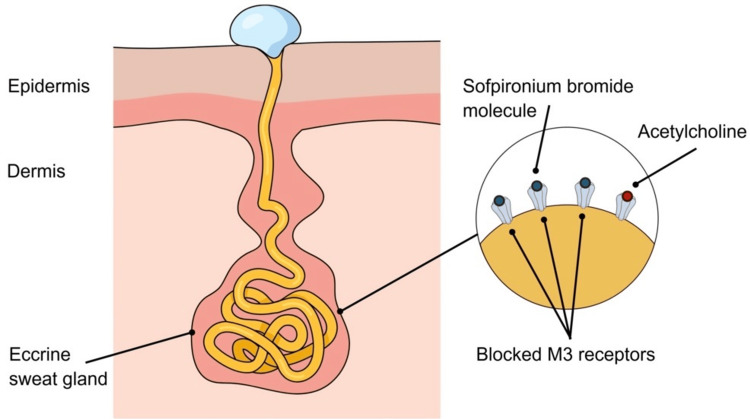
Mechanism of action of sofpironium bromide Figure created by the author using Mind the Graph® (Mind the Graph, São José dos Campos, Brazil).

These findings align with the drug’s underlying mechanism. Sofpironium bromide is a chemically modified ester analog of glycopyrrolate, structurally similar to glycopyrronium bromide. It is a topical anticholinergic agent that acts by selectively inhibiting M3 muscarinic receptors at the eccrine glands, resulting in reduced sweat secretion (Figure 10) [15,18]. It was developed using a retrometabolic drug design approach to provide localized efficacy while minimizing systemic side effects compared with other anticholinergic therapies and is rapidly converted into inactive or less active metabolites upon entering the systemic circulation [15,18].

Consistent with this mechanism, our meta-analysis showed a reduction in sweat production, as reflected by a significant decrease in GSP at the end of treatment. A significant reduction in GSP was observed at week six, but not at week four, suggesting that sofpironium bromide may require a longer treatment duration to demonstrate efficacy. However, Fujimoto et al. reported earlier efficacy, showing a clinically significant improvement in HDSS after one week of treatment, where approximately half of the patients improved from a score of 3 or 4 to 1 or 2 [19]. Nevertheless, these findings cannot be directly compared with ours, as different outcome measures were used. A week-to-week analysis for HDSS could not be performed in the present study due to limited data. In addition, only two studies reported week four data, which may have influenced the overall results.

When stratified by dosage, both the 5% and 15% concentrations demonstrated significant improvements in GSP compared with vehicle, whereas the 10% concentration did not. The 10% subgroup also showed considerable heterogeneity (I² = 73%), which may reflect inconsistent effect estimates between the two available studies. This inconsistency could be attributed to differences in treatment duration and outcome assessment timing. Given the limited number of studies, this pooled estimate should be interpreted cautiously and may not reflect a true lack of efficacy. Moreover, the absence of a significant subgroup difference suggests that differences between concentrations are not clearly established.

In our pooled analysis, discontinuation due to adverse events occurred significantly more often in the sofpironium bromide group than in the vehicle group. Most included studies reported that discontinuations were attributed to anticholinergic effects, such as blurred vision, dry mouth, or mydriasis, as well as local application-site reactions, particularly dermatitis. Although all trials reported some anticholinergic adverse drug reactions (ADRs), only dry mouth reached statistical significance in our analysis, while other systemic effects were infrequent and not significantly different from vehicle. There was no statistically significant difference in the incidence of severe or serious adverse events between groups. Despite the lack of serious safety signals, the temporary effect of sofpironium bromide emphasizes the need to evaluate its long-term safety profile.

The 52-week open-label extension study by Fujimoto et al. reported drug-related ADRs in 39.4% of participants who switched from vehicle to sofpironium bromide and 45.1% of those who received the drug from day one. The most common ADR was application-site dermatitis, which is consistent with our findings showing a significant increase in application-site dermatitis among treated patients. A small number of patients in the long-term study discontinued treatment due to adverse events, including application-site dermatitis, mydriasis, blurred vision, and glaucoma. Most events resolved after discontinuation, except for one case of glaucoma that was lost to follow-up [20].

Most adverse events in our analysis were localized to the application site, such as dermatitis, pain, and pruritus, while only dry mouth appeared to be related to anticholinergic effects. The long-term study also emphasized that dermatitis and irritant reactions were associated with sofpironium use and were likely due to the topical route of administration. These reactions were generally mild and manageable with temporary treatment breaks. The study also reported that anticholinergic adverse events were mild and uncommon [20].

Our analysis showed high heterogeneity in the adverse event outcomes for HDSM-Ax, erythema, and nasopharyngitis. The substantial heterogeneity in HDSM-Ax outcomes may be caused by differences in study settings between the US and Japan, which could influence daily activities and clothing choices, as well as variations in dosing regimens. For erythema, our analysis showed heterogeneity decreased after omitting Kirsch et al., likely due to that study’s broader definition of erythema, which was not limited to application-site reactions. This broader classification may have included unrelated or systemic cases. However, the pooled effect size remained statistically nonsignificant. For nasopharyngitis, heterogeneity decreased to I² = 0% after omitting NCT02336503, and the result became statistically significant. Nevertheless, nasopharyngitis was considered not directly related to sofpironium bromide treatment [13,20].

This study has several limitations. Only five RCTs were included, most with relatively short treatment durations ranging from four to six weeks. Differences in outcome measures also limited the ability to analyze all endpoints. Most subgroup analyses were pre-specified, but the analysis of DLQI was conducted post hoc and was added due to its clinical relevance and data availability. Due to the small number of included studies (n = 5), publication bias could not be formally assessed, and we believe this does not substantially affect the overall interpretation of the results, given the consistency of findings and low heterogeneity across studies. Longer studies are needed to assess long-term efficacy and safety, as well as potential use in other areas. Future research should include direct comparisons with other topical anticholinergic agents, as sofpironium bromide was designed to minimize systemic anticholinergic side effects. Comparative studies with other available hyperhidrosis treatments would further inform clinical decision-making.

## Conclusions

This meta-analysis supports the efficacy of sofpironium bromide in reducing sweat production and improving disease severity and quality of life in patients with primary axillary hyperhidrosis. Treatment was associated with a higher likelihood of achieving clinically meaningful improvement compared with vehicle, particularly with longer treatment duration. However, its use was also associated with a higher incidence of adverse events, most of which were localized and mild, although discontinuation due to adverse events occurred more frequently. Overall, sofpironium bromide represents an effective topical therapeutic option, but treatment decisions should be individualized, balancing clinical benefits against tolerability and patient preference. Further studies are warranted to evaluate long-term safety and optimize treatment strategies.

## Appendices

Appendix 1

Search Strategy

PubMed

("hyperhidrosis"[MeSH Terms] OR "hyperhidrosis"[All Fields] OR "excessive sweating"[All Fields] OR "sweat*"[All Fields]) AND ("sofpironium bromide"[All Fields] OR ("sofpironium"[Supplementary Concept] OR "sofpironium"[All Fields] OR "sofpironium"[All Fields]) OR "BBI-4000"[All Fields] OR "Sofdra"[All Fields] OR "Ecclock"[All Fields])

Embase

('hyperhidrosis'/exp OR hyperhidrosis OR 'excessive sweating'/exp OR 'excessive sweating' OR sweat*) AND ('sofpironium bromide'/exp OR 'sofpironium bromide' OR 'sofpironium'/exp OR sofpironium OR 'bbi-4000'/exp OR 'bbi-4000' OR 'sofdra'/exp OR sofdra OR 'ecclock'/exp OR ecclock)

Cochrane

(hyperhidrosis OR "excessive sweating" OR sweat*) AND ("sofpironium bromide" OR Sofpironium OR "BBI- 4000" OR Sofdra OR Ecclock)
